# Evaluation of Colostral Immunity Against Equine Herpesvirus Type 1 (EHV-1) in Martina Franca's Foals

**DOI:** 10.3389/fvets.2020.579371

**Published:** 2020-11-23

**Authors:** Cristina E. Di Francesco, Camilla Smoglica, Ippolito De Amicis, Federica Cafini, Augusto Carluccio, Alberto Contri

**Affiliations:** Faculty of Veterinary Medicine, University of Teramo, Teramo, Italy

**Keywords:** donkey, Martina Franca breed, serum, colostrum, antibody titer, EHV-1

## Abstract

Eight Martina Franca pregnant jennies were selected in order to evaluate the transfer of colostral antibodies against equine herpesvirus type 1 in their relative foals after immunization with a commercial inactivated vaccine, compared with an unvaccinated group. Samples of serum and colostrums/milk were collected from jennies and foals under study starting from 10 min before and up to 21 days after the foaling. Specific anti-EHV-1 antibody titers were evaluated by means of a serum neutralization test, and the results obtained from both groups were analyzed. The serological titers in the vaccinated jennies was significantly higher (*p* < 0.01). No significant differences were found in the specific time-point intervals in both groups examined (*p* > 0.05). The antibody titers in milk at the time of delivery and subsequent withdrawal (T0 and T1) were very high in both groups, but no significant differences were found between the two groups (*p* > 0.05). In the foal sera, a significant difference was found between foals in the vaccinated group compared with those in the unvaccinated group (*p* < 0.05). Finally, a significant correlation (*p* < 0.05) was observed between the antibody titers found in serum and colostrum of jennies and the foal titers in the first time-point sampling (up to 12 h after foaling). The results confirm a substantial homology in the antibody production compared with other most investigated equids, highlighting the efficacy of the vaccination against EHV-1 of the jennies to ensure the protective immunity to their foals during the first weeks after delivery.

## Introduction

The Martina Franca donkey (MFD) is an asinine breed of great value, native from the Murgia dei Trulli area, in the Apulia provinces of Bari, Taranto, and Brindisi, Italy. It is the largest Italian asinine breed, and because of the imposing size, the stallions of this donkey are widely used in the production of mules, in particular the Martinese mule, generated by the crossing with Murgese horse mares. The MFD population includes 850 donkeys with 25 stallions approved for breeding (1). Over the years, as reported by the FAO Domestic Animal Diversity Information System, the asinine breed of Martina Franca has been identified as endangered. Therefore, any conservation strategies aimed to preserve the health status of the animals and to achieve increases in reproductive success should be carefully considered.

For these reasons, in the past the reproductive characteristics of this breed were investigated in order to ensure the reproductive performances of both females and males of MFD (2, 3). More in detail, the immunity transfer from jennies to foals with particular regard to the immunoglobulin (Ig) G and IgA concentrations in the sera, as well as in milk secretions, was characterized showing a substantial similarity with that reported for horses (4). The higher levels of total IgG can be detected in mammary secretions and serum samples of MFD jennies during the first 10 days after birth, while in donkey foals the serum immunoglobulin concentrations did not show statistically significant differences, although high levels of IgG were observed up to 12 h after delivery (5). It is noteworthy that the antibody transfer from the mother to the foal is essential for the acquisition of passive immunity, and it may be achieved if the intake of colostrum occurs within the first 12–24 h of life (6–8) resulting in a protective action of maternal antibodies into possible external infectious agents. On the other hand, any unfavorable event able to compromise the transfer of colostral immunity in the foal could be critical for the onset of infections during the first 4 weeks of life (9).

Among the pathogens responsible for neonatal diseases in equids, equine herpesvirus-1 (EHV-1) is the more prevalent virus associated with respiratory distress in foals, along with stillbirth, neonatal death, and neurological disease (10). In this regard, the prevention plans for EHV-1-associated diseases require the use of a rigorous vaccination protocol, implemented in both mares and jennies, based on three administrations at the 5th, 7th, and 9th months of gestation (11). In horses, despite the use of the vaccine, the EHV-1 antibody-titers in mares and foals do not appear significantly correlated with the protection, and the fluctuations of the serological response observed in mares and foals are probably due to a silent circulation of the virus among the animals (12).

Even if the passive immunity transfer pattern in donkey foals appears to be similar to that reported for the equine foals, the knowledge about the levels of EHV-1-specific antibodies induced by the vaccination in MF mares and the consequent transfer of them to the foals by colostrum are still lacking, and additional studies could be useful to better characterize the physiology of passive immunity in MFD and to identify the more effective strategies to contrast the neonatal mortality in this threatened breed.

The aim of this study was to compare the titers of colostral antibodies against EHV-1 in serum and colostrums/milk samples collected from both vaccinated and unvaccinated MF mares and their foals starting from the day of the birth up to the 21st day after foaling, in order to verify the kinetic of the passive immunity in both groups under field conditions and to obtain information about the effect of the vaccination on the antibody status of the animals.

## Methods

In the period between 2009 and 2014, *n* = 13 pregnant jennies of the Martina Franca breed (MF) and their respective foals, belonging to the same farm within the Faculty of Veterinary Medicine of Teramo, were investigated.

The jennies were older than 4–5 years, and their body weight was between 396 and 420 kg. During the whole observation period, the animals were kept in external paddocks and exposed to natural atmospheric conditions. Daily, the jennies received standard hay *ad libitum* and commercial horse fodder (2 kg). The Body Condition Score (BCS) of all donkeys was between 3/5 and 4/5 and remained constant for the entire duration of the monitoring.

During pregnancy, *n* = 8 jennies were vaccinated against EHV-1 and EHV-4 using the inactivated Duvaxyn TM EHV-1,4 vaccine (Fort Dodge Animal Health SpA, Italy). The vaccine administrations were carried out at the 5th, 7th, and 9th months of gestation, while the remaining *n* = 5 jennies and all relative foals (belonging to both vaccinated and unvaccinated groups) were not subjected to any administration during the observation period. The recruited jennies showed a regular gestation, and the birth took place without the need for obstetric intervention; the foals appeared clinically healthy at foaling, and they began to take the colostrum without any assistance within the first 2 hours (h) after the foaling.

Serum and colostrum/milk samples were collected from each jenny/foal pair 10 min before foaling up to 21 days postpartum (pp) according to the calendar reported in Table 1 for a total of *n* = 143 colostrum/milk samples and *n* = 286 serum samples.

**Table 1:** Temporal intervals for sera and colostrum sampling from mares and foals under study.

**ID sampling**	**Time-point**
T0	10 min before foaling
T1	1 h after 1st colostrum suck
T2	1 h after 2nd colostrum suck
T3	12 h from foaling
T4	1 day from foaling
T5	2 days from foaling
T6	3 days from foaling
T7	5 days from foaling
T8	7 days from foaling
T9	14 days from foaling
T10	21 days from foaling

All samples were frozen within 2 h of collection and stored at −20°C until laboratory investigations were performed. Before testing, the samples were preheated at 56°C for 30 min to inactivate complement and colostrum was centrifuged at 2,000 rpm for 15 min to remove cellular debris and lipid layer and collect only the liquid portion.

All serum and colostrums/milk samples were tested for serum neutralization (SN) against EHV-1 using RK-13 cells for both viral culture and serum testing (13). Briefly, for each test, 96-well SN plates were prepared with 50 μl of each sample, diluted starting from the 1: 2 dilution up to a dilution of 1: 256, and an equal volume of 100 TCID50 of an EHV-1 vaccine strain propagated on RK13 cells (1 × 105 ml). After incubation of the plates for 45 min at 37°C and 5% CO2, RK13 cells, generally with passage numbers between 85 and 100, were added in the amount of 100 μl (1 × 105/ml) for each well. Appropriate controls for cell viability, virus infectivity, and serum cytotoxicity were included.

All plates were incubated at 37°C and 5% CO2 for 4 days. Then, all plates were examined to detect the cytopathic effect (CPE) of the virus, characterized by cell rounding, formation of syncytia, and detachment of the cell monolayer. The end-point antibody titer was expressed as the reciprocal of the highest dilution of each sample resulting in a complete neutralization of CPE in the cell monolayer, while a sample was defined negative in the presence of viral replication starting from the first 1: 2 dilution.

Data were reported as mean ± standard error of the mean (SEM). Data distribution was tested using the Shapiro–Wilk test. Since the data were not normally distributed, the statistical evaluation was performed after logarithmic transformation.

The evaluation of the data was performed using a generalized linear model (GLM), based on the univariate ANOVA. The biological matrix (maternal serum, milk, and foal serum), time, and vaccination (vaccinated/unvaccinated) were considered as fixed factors. When necessary, a Scheffè test was performed for the *post-hoc* evaluation.

Any correlations between the SN values in the different biological matrices were tested, at various times, by calculating the Pierson correlation coefficient.

In all cases, differences with *p* < 0.05 were considered statistically significant.

The statistical evaluation was performed using the SPSS software version 17 (SPSS Inc., Chicago, IL, USA).

## Results

**Figure 1:**
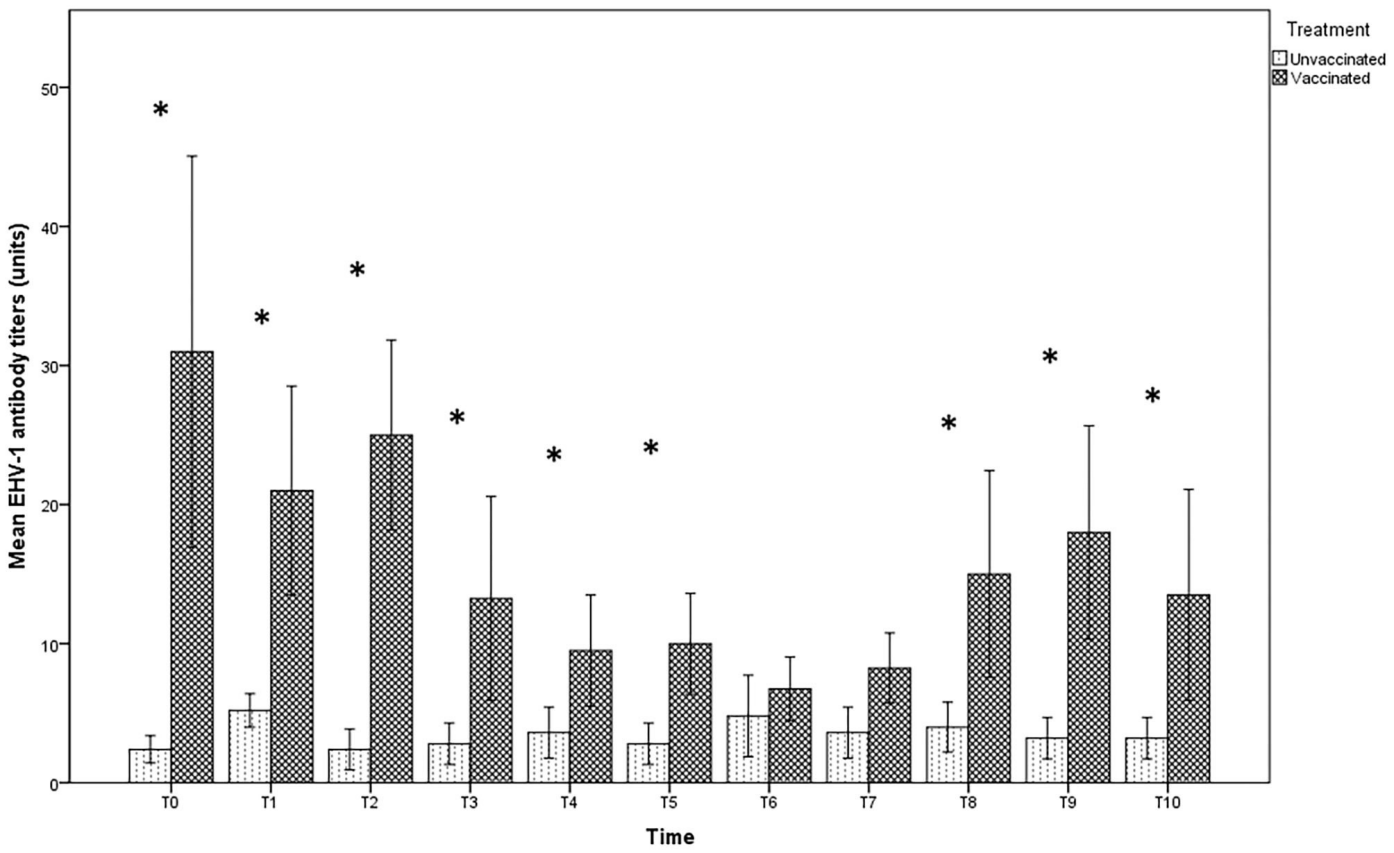
Mean (bar—standard error of the mean) antibody titers against EHV-1 detected by SN test in maternal sera collected during the different time-points of sampling (T0 to T10) from both vaccinated (*n* = 8) and unvaccinated (*n* = 5) jennies under study. At the same time-point, the values of unvaccinated and vaccinated jennies marked with asterisk (*) differ significantly (*p* < 0.05). The titers were expressed as the reciprocal of the highest dilution whit a complete CPE of the cells.

In the group of unvaccinated jennies, the serum antibody titers against EHV-1 were variable from 0 to 1:16; the latter value was obtained only for a serum sample 3 days after delivery (T6). In contrast, for 6 jennies out of 8 vaccinated and at different times of sampling, the antibody titers in the vaccinated group reached values above 1:16, up to 1:128 (Figure 1). The serological titers in the vaccinated jennies was significantly higher (*p* < 0.01). No significant differences were found in the specific time-point intervals in both groups examined (*p* > 0.05).

**Figure 2:**
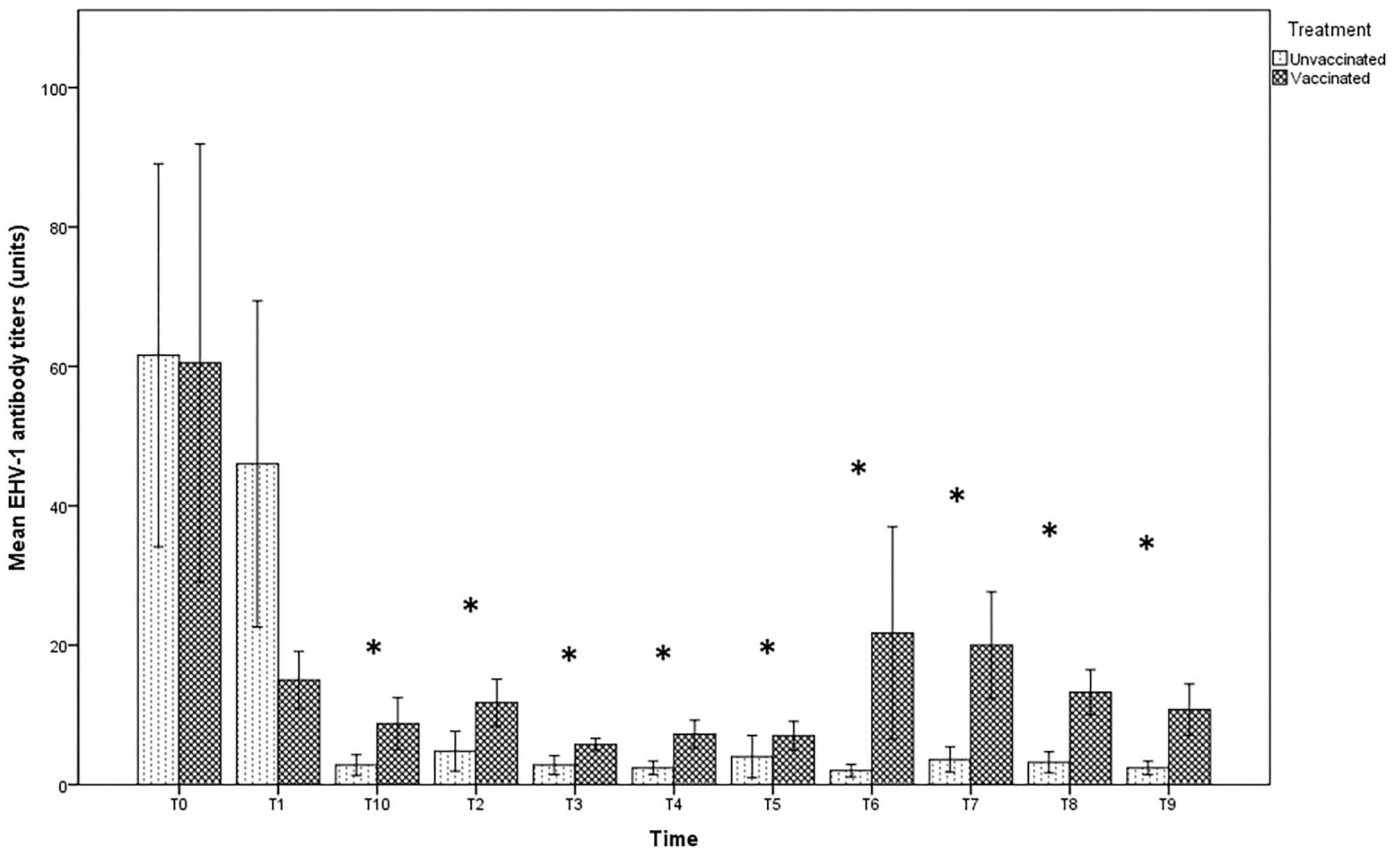
Mean (bar—standard error of the mean) antibody titers against EHV-1 detected by SN test in maternal colostrum/milk samples collected during the different time-points of sampling (T0 to T10) from both vaccinated (*n* = 8) and unvaccinated (*n* = 5) jennies under study. At the same time-point, the values of unvaccinated and vaccinated jennies marked with asterisk (*) differ significantly (*p* < 0.05). The titers were expressed as the reciprocal of the highest dilution with a complete CPE of the cells.

The antibody titer in milk at the time of delivery and subsequent withdrawal (T0 and T1) were very high in both groups, with titers up to 1:128 in unvaccinated jennies and 1:256 in those vaccinated, even if no significant differences were found between the groups (*p* > 0.05). After T2, the values recorded in the milk of vaccinated jennies were significantly higher than those recorded in unvaccinated animals (*p* < 0.05). Indeed, in the group of unvaccinated jennies, the titer decreased reaching the lower values until complete negativity, starting from T2 (after the second sucking). A decrease in antibody concentrations was also found in the group of vaccinated jennies, with antibody titers that returned to increase after T3 until T8 (Figure 2).

**Figure 3:**
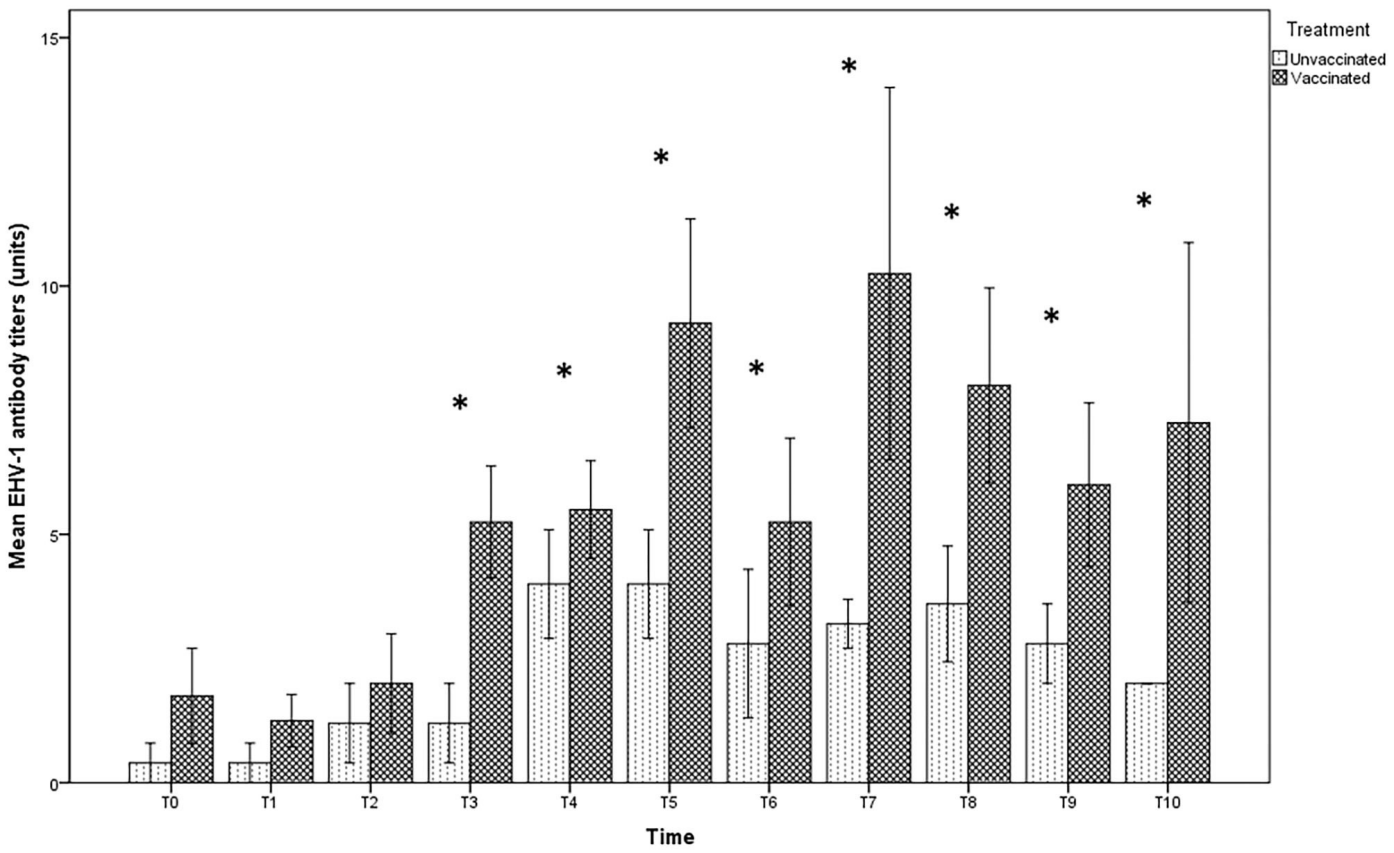
Mean (bar—standard error of the mean) antibody titers against EHV-1 detected by SN test in serum samples collected, at the different time-points (T0 to T10), from foals belonging to both vaccinated (*n* = 8) and unvaccinated (*n* = 5) jennies. At the same time-point, the values of unvaccinated and vaccinated jennies marked with asterisk (*) differ significantly (*p* < 0.05). The titers were expressed as the reciprocal of the highest dilution with a complete CPE of the cells.

In the foal serum, antibody titration showed similar values in both vaccinated (1:8) and unvaccinated groups (1:4), until T2. Twelve hours after foaling, however, a significant increase in the serum titer in the vaccinated group, but not in the unvaccinated one, was found (Figure 3). Statistically significant differences were found between foals in the vaccinated group compared with those in the unvaccinated group (*p* < 0.05) over T3 until the end of the observational period.

The values of the antibody titer found in the mothers were significantly correlated with the foal titer at T1 (*R* = 0.768, *p* < 0.01), while it was significantly correlated with the milk titer at T2 (*R* = 0.729, *p* < 0.01) and with the foal titer (*R* = 0.674, *p* < 0.05) in the same sampling time.

A further significant correlation was found at T9, in which the titer present in the maternal serum was significantly correlated with the antibody titer in milk (*R* = 0.614, *p* < 0.05) and foal (*R* = 0.598, *p* < 0.05). Furthermore, at the same time, a significant correlation was found between the antibody titer in milk and foal (*R* = 0.889, *p* < 0.01).

## Discussion

In this study, the serological investigations were carried out on MF jennies raised in non-experimental field conditions, in open shelters, with the possibility of access to the pasture, and with the presence of other individuals belonging to the same breed and horses. Under these conditions, it is possible to hypothesize a preexisting circulation of HEV-1 among animals, just as it cannot be excluded that other vaccine administrations for the control of the disease have been performed in the previous breeding seasons, considering the age of all jennies subjected to study. Indeed, the infection with HEV-1 is recognized worldwide as endemic among equids, although the vaccination is widely practiced and also thanks to its latency capacity in host cells (14).

In the light of the above information, the presence of anti-EHV-1 antibodies found in the group of jennies that were not subjected to immunization, and persistent throughout the entire study period, even at low levels, is not to be considered unusual. Previous studies have already highlighted that the prevalence of EHV-1 antibody-positive mares and foals did not change significantly when the vaccination is routinely introduced in the horse farms, suggesting a preexisting immunity in the animals consequent to a cycle of infection among the animals (15).

Despite this, the vaccination carried out on a part of the animals has allowed to highlight an overall significant difference in the production of specific antibodies even if this difference is not confirmed by analyzing the individual withdrawal periods. Although a lack of correlation between antibody titer and protection from infection is documented in horses also (16), it is conceivable that the level of antibodies induced by vaccination is able to protect animals from clinical disease and reduce the amount and the duration of viral excretion by the respiratory route. The SN tests carried out starting from milk samples confirmed this difference between vaccinated and unvaccinated subjects, except for T0 and T1 collecting times. In both groups, a significant increase in the concentration of anti-EHV-1 antibodies was observed in the first sampling intervals, confirming the uptake action exerted by the mammary gland in the first hours after delivery, to ensure the massive transfer of immunity to foals through colostrum (17). In the group of vaccinated jennies, moreover, the decrease in antibody levels in the milk appeared to be more gradual and transient, probably favoring a greater intake of specific IgG by the foals in the first 12 h after foaling, when the intestinal absorption of immunoglobulins is greater (18). Similarly, the foal immunity appeared to be influenced by the vaccination of the mothers, with particular emphasis for the samples collected starting from 12 h after foaling. This aspect should be considered when choosing an appropriated vaccination program of the foals, since the presence of high levels of maternal antibody may inhibit the response to the vaccine virus especially for inactivated virus.

The significant correlation observed at T9, 14 days after the delivery, when the transfer of antibodies by the colostrum can be considered completed, appears unusual. Probably, a new introduction of the wild virus strain in both groups under study may have determined this trend, or changes in management and environmental factors resulted in a reactivation of latent infection in stressed mares. Since no movements of new animals or contacts of the foals with other horses were reported during the entire period of the study, the role of stressor factors in the early infection of the foals despite the transfer of passive immunity from vaccinated/seropositive mares should not be ruled out.

In conclusion, this is a first attempt to study the antibody kinetic in MFD after vaccination against EHV-1. The results obtained confirm a substantial homology in the antibody production compared with other well-characterized equids, highlighting the efficacy of the vaccination of the jennies to ensure the protective immunity of the foals during the first weeks after delivery (11, 19). The control of pathogens potentially able to menace the conservation of threatened species such as MFD remains the main tool to preserve the population, and periodic serological surveys of jennies and foals could be considered a useful diagnostic tool to verify the effective immunity reached by the animals after the vaccine administration.

## Data Availability Statement

The raw data supporting the conclusions of this article will be made available by the authors, without undue reservation.

## Ethics Statement

Ethical review and approval was not required for the animal study because In this study the immunological status of donkeys was evaluated after immunization by using commercial vaccine. The antibody titres were obtained from serum samples collected from the animals during the routine clinical investigations in respect of Italian Normative D.Lvo n. 26/2014 (Directive 2010/63/UE).

## Author Contributions

ACa and CD substantially contributed to conception and design of the research. ACo, CD, CS, and ID contributed to acquisition, analysis, and interpretation of the data. ACo, CD, CS, FC, and ID drafted and critically revised the manuscript. All authors ensured that any part of the work is appropriately investigated and resolved and they gave the final approval of the manuscript.

## Conflict of Interest

The authors declare that the research was conducted in the absence of any commercial or financial relationships that could be construed as a potential conflict of interest.
